# Determinants of mother-to-child transmission of hepatitis B virus among pregnant women in southwestern Nigeria

**DOI:** 10.11604/pamj.2024.48.161.41605

**Published:** 2024-08-07

**Authors:** Musa Ayinde, Kehinde Olufemi-Aworinde, Victor Joel-Medewase, Medinat Aliu-Ayinde, Olufemi Aworinde, Adewale Adeyemi

**Affiliations:** 1 Department of Obstetrics and Gynaecology, Faculty of Clinical Sciences, Ladoke Akintola University of Technology, Ogbomoso, Nigeria,; 2 Department of Haematology and Blood Transfusion, Faculty of Basic Clinical Sciences, Ladoke Akintola University of Technology, Ogbomoso, Nigeria,; 3 Department of Paediatrics and Child Health, Faculty of Clinical Sciences, Ladoke Akintola University of Technology, Ogbomoso, Nigeria,; 4 Department of Epidemiology and Community Health, Faculty of Clinical Sciences, University of Ilorin, Ilorin, Nigeria

**Keywords:** HBsAg, HBeAg, HBV DNA, pregnant women, rate of vertical transmission of HBV

## Abstract

**Introduction:**

hepatitis B virus (HBV) infection is a serious public health problem in sub-Saharan Africa and a common cause of liver disease globally. This study aimed to determine the seroprevalence and the rate of mother-to-child transmission of HBV after the age of viability.

**Methods:**

the study was a cross-sectional study that involved 543 eligible consenting pregnant women and newborns of Hepatitis B surface antigen (HBsAg) positive mothers. A one-step rapid HBsAg strip was used to screen eligible patients for HBV infection. Venous blood sample (5mls) was taken from every HBsAg-positive woman for Hepatitis B e Antigen (HBeAg), Hepatitis B e antibody (HBeAb), Hepatitis B core Antibody (HBcAb) and Hepatitis B surface Antibody (HBsAb). In addition, 2mls of cord blood was taken to assay for HBsAg and HBV DNA. Data was analysed using SPSS version 22.

**Results:**

of 543 pregnant women screened, 18 (3.3%) of them were HBsAg-positive with all of them testing negative for HBeAg. HBV DNA was detected in the cord blood of 4 (22.2%) new-borns delivered while 2 (11.1%) tested positive for HBsAg; the above finding indicated that only 4 of the neonates had detectable HBV DNA (>100copies/ml) in their cord blood.

**Conclusion:**

findings from this study demonstrate a low prevalence of HBV infection among pregnant women after the age of viability in Ogbomoso. HBV DNA analysis rather than HBsAg was shown to be more sensitive and specific in determining the risk of intrauterine infections.

## Introduction

Hepatitis B virus (HBV) infection in pregnancy is an important public health concern, particularly in developing countries like Nigeria, HBV is a DNA virus that can be transmitted percutaneously, sexually, or perinatally. Hepatitis B e Antigen (HBeAg) was acknowledged later as a marker of infectivity [1]. Worldwide, about 2 billion people have been infected with HBV, and approximately 400 million people have Chronic Hepatitis B (CHB) infection [2]. The prevalence of HBV infection is relatively high in Africa, which has the second highest number of chronically HBV-infected individuals [3]. The majority of the world's chronic HBV infection carriers, especially children, acquire this infection via the perinatal route. If the infection occurs in the perinatal period, the risk of chronic carriage of HBV infection is about 95% [4].

Hepatitis B infection is endemic in Nigeria, with the prevalence ranging from 9.6% to 18.6% among pregnant women. Currently, about 18 million Nigerians are infected, of which most of them are unaware of their status. The effect of this is an increase in the incidence of complications such as chronic liver disease, cirrhosis, and hepatocellular carcinoma (HCC) [5]. Pregnant women with hepatitis B infection constitute a significant risk to their unborn children, partners, health care providers, and society at large. Mother-to-child transmission (MTCT) is the main route of transmission of hepatitis B virus in areas of high endemicity. MTCT of hepatitis B virus continues to occur despite the interventions of hepatitis B vaccination and immunoglobulin administration in settings where they are practiced. Infants most at risk are those whose mothers have high HBV DNA and produce the protein HBeAg [6]. Various studies in Nigeria have reported high HBV infection rates as well as HBeAg positivity among pregnant women which usually occur during delivery [7].

Detection of specific antibodies and/or antigens in the serum of patients is usually the basis for diagnosis. The most important laboratory test for diagnosis of HBV infection is the detection of HBsAg, which is the first antigen to appear. Antibodies to HBsAg (Anti-HBs) replace HBsAg once the acute infection resolves, and this indicates immunity in about 80% of cases [1]. Anti-HBs also appear after HBV vaccination, but these antibodies could be lost after acute HBV infection, and the patient may thus become susceptible to the disease [2]. Other investigations include the detection of HBeAg, which is an indicator of transmissibility and is replaced by HBeAb, whose presence indicates low transmissibility [2].

The reported prevalence rate of HBV infection among pregnant women in African countries ranged from 6% to 25% [8]. However, in developed countries, such as America and Europe, the prevalence was reported to be 0.1% among pregnant women [6]. The results of these studies confirm the conclusion of the World Health Organization (WHO) that African countries are endemic for HBV infection [8]. The age of viability is the gestational age at which a fetus has a 50% chance of survival in a particular environment. It varies from one country to the other [9]. The World Health Organization (WHO) takes 20 weeks as the age of fetal viability while in Nigeria, the age of viability is taken to be 28 weeks [10]. Since this study was carried out in southwestern Nigeria, 28 weeks was taken as the age of viability. The objective of this study aimed to determine the seroprevalence and the rate of mother-to-child transmission (MTCT) of HBV during pregnancy after the age of viability in southwestern Nigeria and the factors that influence the rate of MTCT.

## Methods

**Study design:** the study was a hospital-based cross-sectional study, conducted between 1^st^ June 2020 to May 31^st^ 2021 in pregnant women after the age of viability.

**Setting:** the study was carried out at the antenatal clinic and labour ward of the Department of Obstetrics and Gynaecology, Ladoke Akintola University of Technology Teaching Hospital (LTH), Ogbomoso, Oyo state, Nigeria.

**Study participants and sampling procedure:** a purposive non-probability sampling technique was used. All eligible and consented pregnant women were recruited into the study from the antenatal clinic and labour ward.

**Research questions:** what is the prevalence of HBsAg positivity among pregnant women after the age of viability? What is the rate of MTCT of hepatitis B virus infection among pregnant women after the age of viability? What are the factors influencing the rate of MTCT of hepatitis B infection among pregnant women after the age of viability?

**Variables and data collection:** participants who consented were interviewed. Pre-test counselling was done, and then the questionnaire was administered to obtain socio-demographic information and relevant history. Blood samples were obtained to screen eligible patients for HBV infection. HBsAg-positive mothers had post-test counselling. Their blood samples were further screened to detect HBeAg, HBeAb, HBcAb, and HBsAb. At delivery, cord blood of neonates of HBsAg-positive mothers was obtained and screened for HBsAg and HBV DNA. The HBsAg-positive mothers were managed according to the unit protocol. This involved delayed artificial rupture of membrane and low-pressure suctioning using an electrical suction machine after delivery. All babies delivered to HBsAg-positive mothers had hepatitis B immunoglobulin within 12 hours of delivery and hepatitis B vaccine on the contralateral thigh before discharge.

**Study size:** the sample size was derived using Fisher's formula [11]:


$$N=\frac{Z^{2}P\left(1-P\right)}{I^{2}}$$

where N is the minimum sample size, z is the z-score that corresponds to the 95% confidence interval (1.96), and p is the expected proportion i.e. prevalence of 7.3% for the disease among pregnant women in Lagos was used [12], and l is the margin of error set at 5%. A sample size of five hundred and forty-three pregnant women was used.

**Statistical methods:** data were entered into the Statistical Product and Service Solutions (SPSS version 22) and analysed. Descriptive statistics was used to summarize the data. The proportions and the corresponding 95% confidence interval (CI) were presented. The frequency table was used for categorical variables, and the Chi-square test was used to test the association between variables. Bivariate analysis for each variable in the study was done and p-value <0.05 for strength of association. Specificity and sensitivity tests were done for both test methods. Multiple regression analysis of the maternal characteristics and neonatal HBV DNA was done.

**Ethical approval:** ethical clearance was obtained from the ethics committee of LAUTECH Teaching Hospital (LTH), Ogbomoso with protocol number LTH/OGB/EC/2020/210. Participant information was treated as strictly confidential and was not used for any unintended purposes.

## Results

**Participants:** five hundred and forty-three (543) subjects were recruited for the study as shown in Table 1. The majority of the respondents were within the age brackets of 20-29 years. More than half of the respondents were within the parity of 0-1 (64%). Nearly all were married with secondary and tertiary levels of education. Only eighteen (3.3%) of the subjects were HBsAg-positive.

**Descriptive/outcome data:** all the eighteen subjects who tested positive for HBV were negative for HBeAg. However, most of them (>70%) tested positive for Anti-HBsAg, Anti-HBeAg and anti-HBc antibodies (Table 2). The majority of the respondents delivered through spontaneous vaginal delivery. Most of the respondents (66.7%) had spontaneous rupture of membranes with most having less than 4 hours interval between membranes rupture and delivery. Most of the respondents (88.9%) delivered at term with labour duration of less than 12 hours (Table 2).

**Table 1:** socio-demographic characteristics of the respondents (n=543)

Variables	Frequency	Percentage (%)
**Age (years)**		
<20 years	63	11.6
20-29 years	276	50.8
30-39 years	141	26.0
≥40years	63	11.6
**Parity**		
0	180	33.1
1	168	30.9
2	95	17.5
3	84	15.5
4	8	1.5
≥5	8	1.5
**Marital Status**		
Single	3	0.6
Married	540	99.4
**Educational Status**		
No formal education	18	3.3
Primary School	9	1.7
Secondary School	116	21.4
Tertiary Education	418	73.7
**Religion**		
Islam	152	28
Christianity	391	72

**Table 2:** HBV serology profile and delivery history of HBV positive subjects (n=18)

Variables (n=18)	Detection	Frequency	Percentage (%)
HBeAg	Positive	0	0
	Negative	18	100
Anti-HBc	Positive	13	72.2
	Negative	5	27.8
Anti-HBeAg	Positive	15	83.3
	Negative	3	16.7
Anti-HBsAg	Positive	14	77.8
	Negative	4	22.2
**Delivery history**	**Variable**	**Frequency**	**Percentage (%)**
Spontaneous vaginal delivery	Episiotomy given	5	27.8
	Episiotomy not given	8	44.4
Caesarean section	Emergency	3	16.7
	Elective	2	11.1
Rupture of membrane (RM)	Spontaneous	11	61.1
	ARM	7	38.9
Interval between RM and delivery	<4 hours	12	66.7
	>4 hours	6	33.3
Gestational age at delivery	<37 weeks	2	11.1
	>37 weeks	16	88.9
Birth weight (kg)	<2.5	1	5.5
	≥2.5	17	94.5
Apgar score (at 5min)	<7	2	11.2
	≥7	16	88.8
Duration of Labour	<12 hours	12	66.7
	>12 hours	6	33.3
**Rate of Intrauterine Infection**	**Detection**	**Frequency**	**Percentage (%)**
Cord blood for HBsAg	Positive	2	11.1
	Negative	16	88.9
Cord blood for HBV-DNA	Positive	4	22.2
	Negative	14	77.8

**Main results:** Table 3 shows that the rate of mother-to-child transmission of HBV infection among neonates of HBsAg-positive mothers delivered after the age of viability was 11.1% using HBsAg and 22.2% using HBV DNA (>100 copies/ml). It was found that 4 (22.2%) out of the 18 cord blood samples gotten from neonates of HBsAg-positive mothers were positive for HBV DNA while 2 (11.1%) were positive for HBsAg. This was statistically significant (p<0.05). The sensitivity and specificity of HBV DNA for detecting mother-to-child transmission of hepatitis B infection were 67.1% and 87.5% respectively while the sensitivity and specificity of HBsAg for detecting mother-to-child transmission of hepatitis B infection were 32.9% and 12.5% respectively (Table 3, Table 4, Table 5).

**Table 3:** association between cord blood detection of HBV DNA and HBsAg positivity, its sensitivity and specificity

Baby (n = 18)	HBsAg positive	HBsAg negative	Sensitivity	Specificity	p-value
HBV-DNA detected	2	2	67.1%	87.5%	0.001a
HBV-DNA not detected	0	14	32.9%	12.5%	0.031b

**Table 4:** relationship between delivery history and MTCT

Delivery history	Positive (n=4) Frequency %		Negative (n=14) Frequency %		p-value
Duration of labour	0	0.0	2	11.1	**0.005**
Time interval between rupture of membrane and delivery	1	5.6	2	11.1	**0.003**
EGA at delivery	0	0.0	1	5.6	0.222
Birth weight	0	0.0	3	16.7	0.568
Elective	0	0.0	1	5.6	0.301
Emergency	1	5.6	4	22.2	0.540
SVD	0	0.0	1	5.6	0.453
APGAR score (5 minute)	2	11.1	0	0.0	0.131
HbeAg	0	0.0	18	100	0.211

**Table 5:** relationship between maternal characteristics and neonatal HBV DNA

Delivery history	Neonatal HBV DNA titre (Log10 copies /ml)				
	Regression Coefficients	t-value	p-value	95%CI	
				Lower	Upper
Duration of Labour	0.152	14.57	0.001	0.9503	1.2719
Time interval between rupture of membrane and delivery	-.029	3.313	0.005	2.0155	9.0845
EGA at delivery	1.113	11.466	0.222	7.5941	11.1202
Birth weight	.783	13.626	0.722	1.3617	1.8606
Elective (C/S)	1.803	7.827	0.401	-.0240	.3574
Emergency (C/S)	1.274	2.244	0.640	.3667	1.5222
SVD	1.262	9.454	0.453	1.5105	2.3784
APGAR score (5minute)	.396	2.113	0.852	1.6426	2.0240

CI: Confidence interval

**Other analysis:** multiple regression analysis of the maternal characteristics and neonatal HBV DNA presence showed that an increase in the duration of labour and time interval between membrane rupture and delivery were associated with an increase in neonatal HBV DNA titre (p<0.005) (Table 3).

## Discussion

**Key results:** five hundred and forty-three women were recruited for this study. Most respondents were married young adults. This was similar to the findings of Aworinde *et al*. [13,14]. Eighteen of the women recruited tested positive for HBsAg, putting the prevalence of hepatitis B infection at 3.3%. This indicates a moderate prevalence of hepatitis B in our environment and does not support the inclusion of Nigeria by WHO as a high-endemic region with a prevalence greater than 8% [15]. The moderate prevalence in this study could be a result of the implementation of the national HBV infection prevention strategic plan. The strategic plan may have led to the reduction in the burden of endemicity of HBV infection and ultimately the rate of mother-to-child transmission. The national HBV infection prevention strategic plan includes routine screening of pregnant women and administration of passive immunoglobulin to all neonates of HBsAg-positive women in addition to active hepatitis B immunization to all newborns irrespective of the status of the mother. Another factor that might be responsible for the low prevalence may be the fact that the study was a hospital-based study involving only booked women whose pregnancy had reached the age of viability. Similar studies done in Osogbo and Makurdi reported low prevalence rates of 2.8% and 3.9% respectively, while higher values were recorded by other researchers [16-19].

This research tested for the presence of HBsAg and HBeAg in maternal blood as well as for both HBsAg and HBV DNA in the cord blood of neonates born to mothers who were HBsAg positive. The absence of HBeAg in all the respondents was similar to the findings in other African studies [4,14,20]. This could be due to the predominance of HBeAg-negative chronic HBV linked to HBV genotype D which is prevalent in Africa [21,22]. Maternal HBV DNA was not done in this study due to the negative HBeAg status of all the studied patients which indicates a low level of maternal viraemia and infectivity. However, other researchers such as Obi *et al*. [23] in Port Harcourt and Olaleye *et al*. in Ile-Ife noted a significant relationship between the maternal HBeAg, HBV DNA >10^7^ IU/ml and the risk of intrauterine infections [15,23]. These findings support a significant relationship between negative HBeAg, suspected low level of maternal viral load and lower risk of intra-uterine infection which was noted in our findings.

Multiple sexual partners, change of husband, previous history of blood transfusion, history of scarification and sharing of hair-parting needles were noted to be significantly associated with HBV infections among pregnant women recruited in our study. Similar findings were also noted by other researchers [23-27]. HBV is an organism that thrives in blood and other body fluids; hence, its transmission through the above routes is not surprising.

The incidence of mother-to-child transmission of HBV varies from 2.1 to 50% as reported by different researchers [28]. The vertical transmission risk of 22.2% was found in this study using HBV DNA and 11.1% using HBsAg with specificity of 87.5% and 12.5% respectively. Zhang and his colleagues found a lower incidence of 17.1% when using HBsAg compared to 41.5% on using HBV DNA [28]. Olaleye *et al*. reported an incidence of 24% using HBsAg and 72% using HBV DNA in Ile Ife [15]. The risk of intrauterine infection noticed in this study appeared lower when compared with the two studies above. The reason could be a result of the fact that most patients studied were in the reactivation phase with negative HBeAg which inferred a low level of maternal viral load which could account for the low risk of perinatal transmission as found in this study.

A negative maternal HBeAg was suspected to be the reason for the low risk of intrauterine infection reported in our study when compared with other studies, this is in line with the findings of Olaleye *et al*. and Xu *et al*. on a direct significant relationship between very high maternal HBV DNA levels and neonatal risk of intra-uterine infections [15,28]. There was a statistically significant association between the risk of MTCT of HBV DNA, prolonged labour and time interval between rupture of membrane and delivery greater than 4 hours. Olaleye *et al*. and Lavanchy *et al*. also had similar findings in their studies [15,29]. However a study done by Mimi and Cheung in Hong Kong found the mode of delivery to be significantly associated with the risk of MTCT of HBV DNA unlike in this study [30].

**Limitations:** none of the subjects studied was HBeAg positive so its association with HBV DNA core antigen in maternal-to-child transmission could not be assessed.

## Conclusion

Findings from this study demonstrate a low prevalence of HBV infection among pregnant women after the age of viability in Ogbomoso. HBV DNA analysis rather than HBsAg was shown to be more sensitive and specific in determining the risk of intrauterine infections.

### What is known about this topic

- *Pregnant women with hepatitis B infection constitute a significant risk to their unborn children with infants most at risk being those whose mothers have high HBV DNA*.

### What this study adds

- *The prevalence of hepatitis B in this study does not corroborate the previous inclusion of Nigeria by WHO as a high endemic region with a prevalence greater than 8%;*
- *The rate of MTCT is low in the absence of HBeAg*.
